# Dual-Action Icariin-Containing Thermosensitive Hydrogel for Wound Macrophage Polarization and Hair-Follicle Neogenesis

**DOI:** 10.3389/fbioe.2022.902894

**Published:** 2022-06-27

**Authors:** Ying-Ying Teng, Ming-Li Zou, Si-Yu Liu, Yuan Jia, Kai-Wen Zhang, Zheng-Dong Yuan, Jun-Jie Wu, Jun-Xing Ye, Shun Yu, Xia Li, Xiao-Jin Zhou, Feng-Lai Yuan

**Affiliations:** ^1^ Institute of Integrated Chinese and Western Medicine, The Hospital Affiliated to Jiangnan University, Wuxi, China; ^2^ Wuxi Clinical Medicine School of Integrated Chinese and Western Medicine, Nanjing University of Chinese Medicine, Wuxi, China

**Keywords:** icariin, wound repair, bone morphogenetic protein 4, macrophage polarization, hair-follicle regeneration

## Abstract

Bone morphogenetic protein (BMP) pathway is essential for M2 macrophage polarization and hair-follicle neogenesis. Icariin, a flavonoid derived from *Epimedium*, is a mediator of the BMP pathway. Here, we develop a hydrogel formulation functionalized with icariin for regulation of macrophage polarization to accelerate wound healing and hair-follicle neogenesis. Compared to skin defects without icariin treatment, those treated with icariin+PEG hydrogel healed faster and had new hair follicles. Results *in vivo* showed that icariin+PEG hydrogel induced a higher level of M2 phenotypic transformation of macrophages. Moreover, icariin+PEG hydrogel significantly accelerated wound-repair process by reducing the invasion of inflammation, excessive deposition of collagen, immoderate activation of myofibroblasts, and increasing the regeneration of hair follicles. Furthermore, studies *in vitro* demonstrated that the icariin+PEG hydrogel induced macrophages to polarize to the M2 phenotype and dermal papilla cell to hair follicles. Finally, molecular analysis demonstrated that the icariin+PEG hydrogel increased the expression of BMP4 and Smad1/5 phosphorylation in skin wounds. These results demonstrate the therapeutic potential of icariin-containing thermosensitive hydrogels for inducing M2 macrophage polarization to accelerate wound healing and promote hair-follicle neogenesis by regulating the BMP pathway.

## Introduction

Skin, the largest organ of the human body, plays a vital role in protecting the body from environmental and microbial invasion (Zhao et al., 2017). However, skin injuries, especially chronic wounds, burns, and infected wounds, when not cared for appropriately, can make the healing process difficult or promote the formation of pathological scars, causing dysfunction of the protective role of the skin and burdening an already overloaded health care system (Zhu et al., 2017; Zhong et al., 2019). Scar tissue comprises cells (mainly fibroblasts) and unorganized collagen and elastic fibers. They lack functional skin accessories (sweat glands, sebaceous glands, and hair follicles). Hypertrophic scar is often symptomatic and causes itching, burning, pain, sensation, and thermoregulation disability (Stoddard et al., 2014). Although most of the drugs and bio-activators, wound dressing, and autologous skin grafting can promote wound healing well, regenerating the skin with complete appendages remains challenging. Therefore, a new treatment method should be developed for faster wound healing with hair-follicle regeneration.

Wound healing is a complex process. It has three major overlapping phases: inflammation, formation of new tissues, and remodeling (Takeo et al., 2015). The immune response in the early stage of wound healing plays a crucial role in tissue regeneration. Although inflammation at the site of tissue injury is necessary for initiating the healing response, the elimination of inflammation is also essential for promoting the healing process and restoring tissue integrity (Forbes and Rosenthal 2014). Macrophages are one of the most important inflammatory cell types involved in wound healing (Funes et al., 2018). Polarized macrophages can be activated according to their functions (Jiménez-García et al., 2018). M1 macrophages have a proinflammatory role and mainly secrete proinflammatory factors, while M2 macrophages can reduce inflammation and perform tissue-repair functions. Timely transformation of M1 macrophages to M2 macrophages (the anti-inflammatory phenotype) has great applicability in regenerative medicine (Sindrilaru et al., 2011; Miao et al., 2016; Li et al., 2021). If the skin appendages (hair follicles, sweat glands, and sebaceous glands) are completely destroyed, they cannot be fully regenerated, resulting in a scar (Murawala et al., 2012). Many studies have shown that hair follicles possibly regenerate mesenchymal cells during wound healing (Rippa et al., 2019; de Groot et al., 2021). Application of drugs and different drug-delivery systems in the healing process has been investigated. For example, prostaglandin E2 is used to regulate the phenotype of macrophages to reduce inflammation and promote wound healing (Zhang et al., 2018). Parker *et al.* have produced large-scale fibronectin nanofibers to repair the dermal papilla and recruit basal epithelial cells to promote the regeneration of hair follicles (Chantre et al., 2018). However, none of these strategies restore the skin tissue to its original form with a dual synergistic function, i.e., suppression of inflammation and promotion of hair-follicle regeneration during the wound healing process.

Recent studies have revealed that bone morphogenetic protein 4 (BMP4) is a promising option for wound healing because it can regulate cell proliferation and differentiation, macrophage polarization, stem-cell self-renewal, and embryonic development (Tong et al., 2015). Interestingly, a recent study has confirmed that the enhanced BMP signaling in myofibroblasts is related to hair-follicle regeneration and might promote wound healing (Plikus et al., 2017). Moreover, the participation of BMP4 can promote the proliferation and migration of dermal papilla cells and induce hair-shaft differentiation (Daszczuk et al., 2020). Interestingly, in acute lymphoblastic leukemia, BMP4 can upregulate the expression of interleukin (IL)-10 and promote the polarization of M1-like macrophages to the M2 phenotype (Zylbersztejn et al., 2018). Therefore, a material that is easy to source and able to modulate skin wound healing processes may be a potential solution to accelerate this process and reduce scar tissue formation by regulating BMP4 signaling.

Icariin, an extract from the traditional Chinese medicine Herba Epimedii, can increase the expression of BMP4 pathway components, enhance BMP4 signal transduction, and accelerate wound healing (Liu et al., 2018; Mi et al., 2018; Singh et al., 2019; Owen et al., 2020; Xie et al., 2020). Its remarkable pharmacological and biological effects, such as anti-inflammatory, antitumor, and neuroprotective effects, have already been confirmed (Shen and Wang 2018; He et al., 2020). Although Mi Bobin el. studied the effect of icariin on wound healing, they only focused on the role of keratinocytes (Mi et al., 2018), ignored the potential roles of macrophages and hair follicles and their possible mechanisms in skin wounds. Medical biomaterials can control the release of cytokines and drugs in time and space to simulate the dynamic changes of signals during normal tissue regeneration (Sun et al., 2018; Xu et al., 2018). However, the efficacy of icariin with hydrogels for accelerating wound healing and hair-follicle neogenesis by activating the BMP4 signaling pathway has not yet been elucidated. We hypothesized that treatment concepts based on mediation strategies of the BMP4 pathway in wounds could have a therapeutic potential. Herein, we develop a novel thermosensitive hydrogel drug-delivery system by encapsulating icariin with a poly (lactic acid-co-glycolic acid)–poly (ethylene glycol)–poly (lactic acid-co-glycolic acid) (PLGA–PEG–PLGA) triblock copolymer-based hydrogel. We also investigate its effects on macrophage polarization both *in vitro* and *in vivo* as well as on the regeneration of hair follicles. The findings of this study may help improve the wound healing process.

## Materials and Methods

### Hydrogel Preparation

DL-lactide, glycolide, and polyethylene glycol (molar ratio: 2:2:1, Guidechem Chemical, China) were added to a three-necked flask, followed by the addition of 0.5% stannous octoate as a catalyst (Guidechem Chemical, China). The mixture was then repeatedly ventilated with nitrogen and vacuumed to remove trace amounts of moisture and oxygen. Next, under normal pressure, the tube was filled with nitrogen and then heated to 160°C under magnetic stirring for 8 h to obtain the PLGA–PEG–PLGA copolymer. The polymer was dissolved in dichloromethane and precipitated using petroleum ether to obtain a purified product. The polymer was then vacuum-dried to a constant weight. Finally, a 20 wt% solution of the polymer in deionized water was made and used as a temperature-sensitive hydrogel. The thermosensitive hydrogel was placed in a 1.5 ml EP tube, and a specific amount of icariin (489-32-7, Sigma-Aldrich) was dispersed in the thermosensitive hydrogel, which was completely dissolved by shaking in a 4°C refrigerator.

### Hydrogel Morphology

The morphology of the hydrogel was observed using a cryo-scanning electron microscope (Cryo-SEM, Quorum, United Kingdom). The samples were frozen by liquid nitrogen and put onto the cold table of an electron microscope (the temperature of which can reach −185°C) through the freezing transmission system for observation.

### Rheological Analysis

Rheological analysis of the hydrogels was performed on a rheometer (Mars 40, Thermo Fisher Scientific, Germany) equipped with a 40 mm diameter parallel plate geometry. The hydrogel precursor solutions were pipetted between the parallel plates with a gap of 0.5 mm. Then the Oscillatory rheological measurements were carried on as the temperature of the plate was heated up from 4 to 37°C at a heating rate of 1°C min-1 and balanced at 37°C for 600 s to measure the storage modulus (G′) and loss modulus (G″). In addition, cycling three times between 1 and 1,000% was conducted to test its self-healing property.

### Release of Icariin

After coagulation in a 15-ml centrifuge tube, 1 ml of the icariin+PEG hydrogel was shaken (37 °C, 60 rpm) with phosphate-buffered saline (PBS) (10 ml). Subsequently, 5.0 ml of the released medium was removed after certain time intervals (0.5, 1.5, 3, 5, 8, 12, 16, 20, 24, 32, 40, and 48 h) and replaced with 5.0 ml of fresh buffer. An ultraviolet-visible spectrophotometer (LAMBDA 35, PerkinElmer) set at a wavelength of 283 nm was used to determine the concentration of icariin in the released medium, and quantitative analysis was performed with the standard curve of previously prepared buffer solutions.

### Cell Lines and Cell Cultures

The human monocytic cell line THP-1 was used to detect the polarization of macrophages *in vitro*. 5.0 × 104 cells per well were seeded in 6-well plates and treated by 100 ng/ml PMA for 24 h at 37°C. This cultivation method used herein was designed based on previous studies (Borchert et al., 2021). After the cells adhered, the culture medium was replaced with fresh medium, and the effect of the hydrogel on macrophages was observed by adding different treatments (PBS, PEG hydrogel, icariin only, icariin+PEG hydrogel) above the transwell chamber. The morphology of the cells was photographed with a microscope (Olympus Corporation, Tokyo, Japan).

Human hair dermal papilla cells (HDDPCs) (ZQY002, Zhongqiaoxinzhou Biotech, Shanghai, China) were cultured in Mesenchymal stem cell medium with 5% fetal bovine serum (FBS), 1% mesenchymal stem cell growth factor, 100 U/mL penicillin, and 100 μg/ml streptomycin. The cells were cultured at 37°C and 5% CO_2_ and were serially passaged at 85–95% confluence.

### Cytotoxicity Assay

The biocompatibility and cytotoxicity of the hydrogel were tested by both a cell counting kit-8 (CCK-8) assay and live/dead cell staining (Beyotime, China).

### Immunofluorescence Staining

The influence of the hydrogel on the macrophage phenotype and inflammatory response was detected by immunofluorescence staining, according to methods described previously (Feng et al., 2020). The fluorescence images were taken by an inverted fluorescence microscope. Primary antibodies against CD206, tumor necrosis factor-alpha (TNF-α), cytokeratin, CD68, CD31, and Ki67 as well as secondary antibodies for fluorescence staining were purchased from Abcam (Cambridge, United Kingdom). Goat anti-rabbit (H + L) horseradish peroxidase secondary antibody was purchased from Bioworld Technology (St. Louis, MO, United States).

### Western Blotting

Western blotting was performed to explore M2 polarization of macrophages and the level of skin fibrosis. The following antibodies were used in the western blotting assays: alpha-smooth muscle actin (α-SMA, 1:100, ab8211, Abcam); IL-10 (1:100, ab34843, Abcam); type I collagen (Col Ⅰ, 1:200, ab260043, Abcam); IL-6 (1:500, ab6672, Abcam); cytokeratin 17 (1:500, ab109725, Abcam); TNF-α (1:1000, ab6671, Abcam); arginase-1 (1:500/1:50, #93668, Cell Signaling Technology); CD206 (1:1000, #PA5-114310, Invitrogen); Smad1/5 (1:500, bs-2973R, Bioss Antibodies); phospho-Smad1/5 (1:1000, bs-3418R, Bioss Antibodies); BMP4 (1:500, bs-1374R, Bioss Antibodies); and GAPDH (1:10000, ab181602, Abcam).

### Reverse Transcription–Polymerase Chain Reaction (RT-PCR)

A real-time quantitative RT-PCR system (QuantStudio 3, Thermo, United States) was used to detect changes at the gene level. The sequences of the primers are listed in Supplementary Table S1.

### Flow Cytometric Analysis

After the treatment, 1 × 10^6^ cells belonging to the control group, icariin group, and IL-4 group were incubated with 2 μL of FTTC anti-human CD206 antibody (321104, Biolegend) and 2 μL of APC anti-human CD68 antibody (333809, Biolegend) diluted in a staining buffer for 30 min on ice in the dark. Next, the cells were washed twice with the staining buffer to remove excess antibodies, followed by their resuspension in 500 μL of the staining buffer. The stained cells were analyzed by flow cytometry (BD FACSCalibur, San Jose, CA, United States). The flow cytometry data was analyzed by FlowGo.

### Animal Model for Dermal Wound Healing

Sterile ophthalmic scissors were used to create skin full-thickness wounds of 1-cm diameter in the back of male C57BL/6 mice (8–12 weeks) deep to the fascia. The mice were randomly divided into three groups: those treated with 0.9% saline, those treated with PEG hydrogels, and the rest treated with icariin-loaded PEG hydrogels. The hydrogels were directly injected onto the surface of each wound, which was then covered with a 3M Tegaderm^TM^ film and secured with a medical bandage. A digital camera was used to take photos of the wound at 0, 3, 7, 10, and 14 days after the treatment. ImageJ was used to analyze the wound-healing rate. All procedures were approved by the Experimental Animal Committee of Jiangnan University, China.

### Histological Analysis

Hematoxylin and eosin staining, Masson’s staining, and immunofluorescence staining (Beyotime, China) were performed on the skin tissue slices to analyze the wound condition, including the healing speed, fibrosis, and regeneration of the skin appendages. The images were analyzed by ImageJ software.

### Cell Migration Assay

Cells were plated at a density of 1×10^5^/well in 6-well plates. When the transfected cells reached 100% confluence, a sterile micropipette tip was used to create a scratch. The cells were washed with PBS, and minimal medium was added. Cell migration was observed and imaged at 0 h and after incubation for 6 and 12 h at 37°C. Cell migration was analyzed using ImageJ software.

### Statistical Analysis

Statistical analysis was performed using GraphPad Prism 8.0.1 software (GraphPad Software Inc., San Diego, CA, United States). Data were expressed as the mean ± standard error of the mean. Statistically significant differences between the groups were assessed by analysis of variance or the two-tailed Student’s *t*-test. For all tests, *p < 0.05, **p < 0.01, and ***p < 0.001 were considered statistically significant.

## Results

### Characteristics of icariin+PEG Hydrogel

**FIGURE 1:**
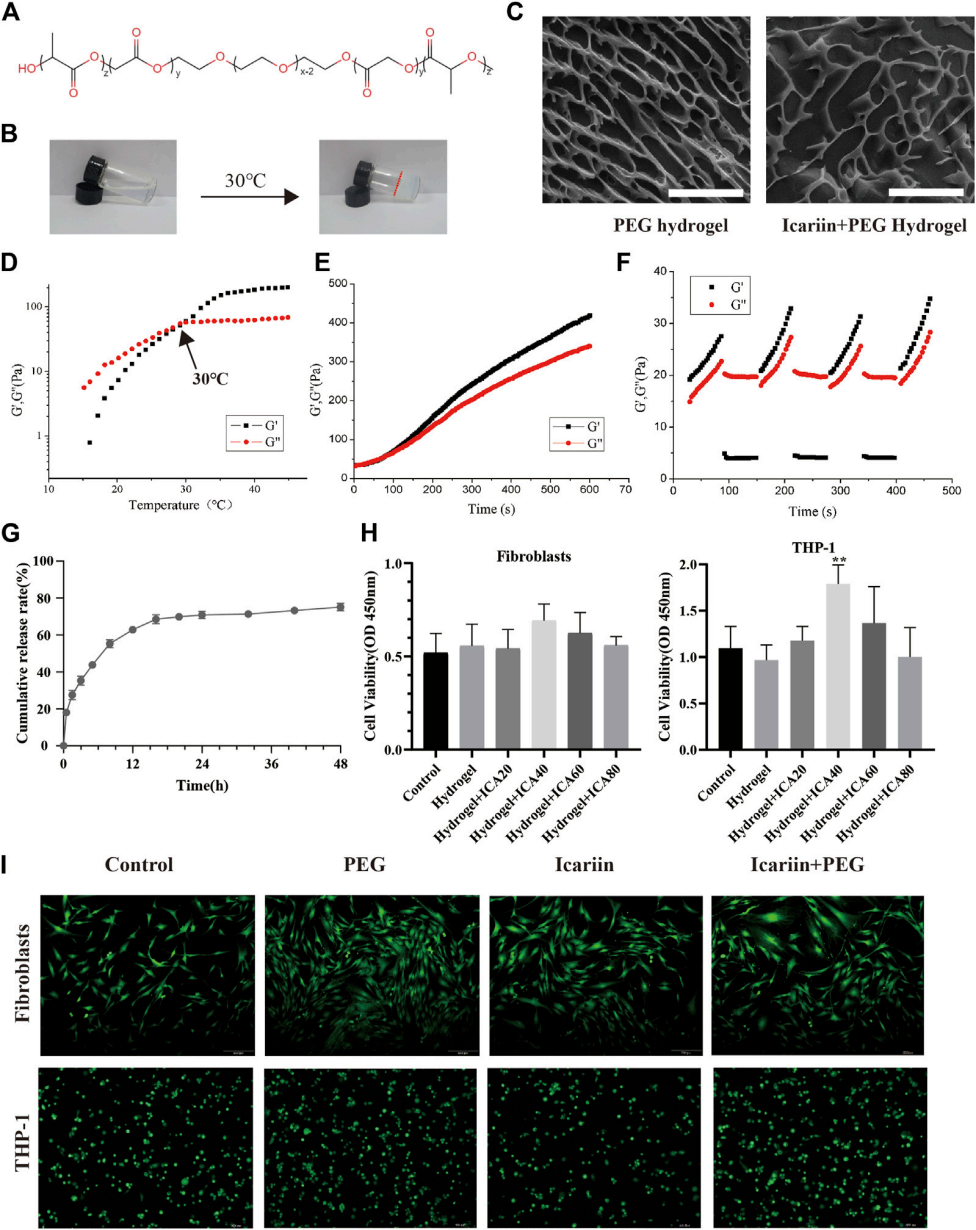
Characterization and biocompatibility of the PEG hydrogel. **(A)** Chemical structure of the PEG hydrogel. **(B)** PEG hydrogel solution gelled at 30°C. **(C)** Cryo-scanning electron microscopy images of PEG and icariin+PEG hydrogels. Scale bar, 30 μm. **(D)** Temperature dependence of the storage modulus (G′) and loss modulus (G″) of the PEG hydrogel. **(E)** Gelation time of the PEG hydrogel at 35°C. **(F)** G′ and G″ of the PEG hydrogel when circulated three times between strain of 1 and 1,000%. **(G)** Release profile of icariin from the icariin+PEG hydrogel by UV spectrophotometry. **(H)** Cell viability on human fibroblast cells and THP-1 macrophages with hydrogel extracts of different compositions for 48 h. ICA20: 20 μg/ml icariin, ICA40: 40 μg/ml icariin, ICA60: 60 μg/ml icariin, ICA80: 80 μg/ml icariin, **p < 0.01 *vs*. the control. **(I)** Fluorescence microscopy of fibroblasts and macrophages was performed with live/dead cell staining. Live cells emit green fluorescence, while dead cells emit red fluorescence.

The hydrogel was fabricated using DL-lactide, glycolide, and polyethylene glycol (Figure 1A). The hydrogel was thermosensitive and could be transformed from a liquid state to a hydrogel at 30°C (Figure 1B). The microstructure of the hydrogel was observed by cryo-scanning electron microscopy. As shown in Figure 1C, both the PEG and icariin+PEG hydrogels presented interconnected three-dimensional networks with a uniform pore size distribution, indicating that icariin was evenly distributed inside the PLGA–PEG–PLGA network, without forming agglomerates. To further verify the performance of the PEG hydrogel upon a temperature change, an oscillating rheological test was conducted. At 15–45°C, the storage modulus (G′) of the hydrogel gradually exceeded the loss modulus (G″), indicating that the sol–gel changes at the critical temperature of 30°C (Figures 1D,E). Therefore, the refrigerated icariin+PEG hydrogel can be injected into the wound and cover wounds of any shape and quickly become a colloid when heated to 30°C, which is close to the surface temperature of the human body. The self-healing ability of the hydrogel dressings was also confirmed (Figure 1F). After conducting three steps of strain cycles during the rheological analysis, the quick drop in the values of G′ and G″ at high strain (1,000%) and their rapid recovery under low shear strain (1%) indicated that the hydrogel has a good shear-thinning ability and self-healing properties. The hydrogels merged without any visible interface when solutions of two different colors were incubated for 30 min, indicating that the hydrogel has a good self-healing ability (Supplementary Figure S1B). The drug-release efficiency of the hydrogel was evaluated by monitoring the concentration of icariin in the supernatant. Figure 1G shows that the release curve was relatively stable and gradually increased, reaching a peak at 24 h. The CCK-8 results showed that the drug-loaded gel extract at the optimal concentration of 40 μg/ml could promote the proliferation of macrophages and did not exert any significant effect on the growth of fibroblasts (Figure 1H). To further study the biocompatibility of the hydrogel, transwells were used to co-culture the hydrogel and cells. A representative live/dead cell staining experiment is shown in Figure 1I. The cells incubated with PBS, icariin, PEG hydrogel, and icariin+PEG hydrogel showed shining green fluorescence; red fluorescence could scarcely be seen in the images, implying that there was little cell death and a good biocompatibility.

### Icariin+PEG Hydrogel Accelerates *in vivo* Wound Repair Process

**FIGURE 2:**
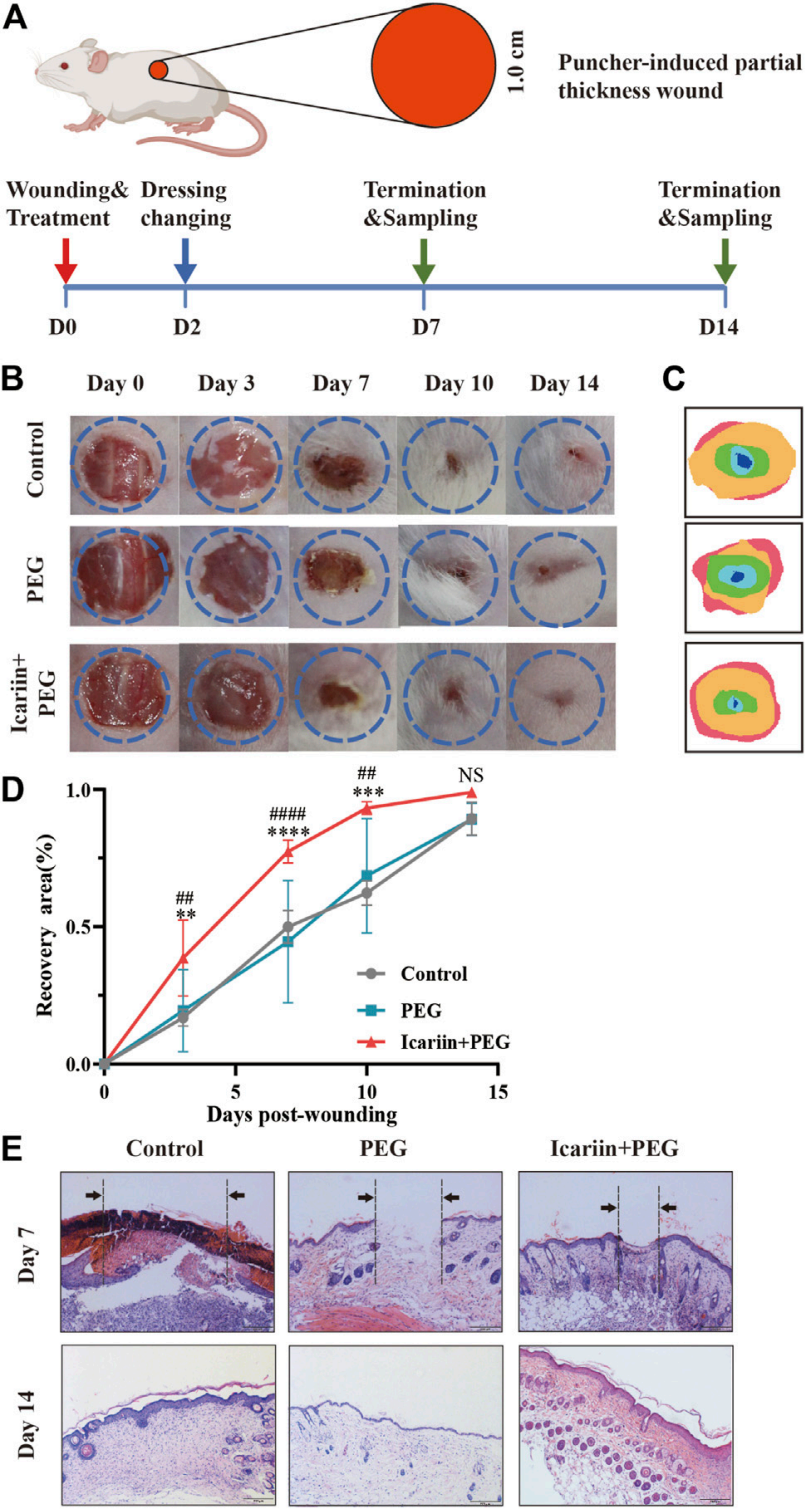
Icariin+PEG hydrogel speeded up skin repair. **(A)** Schematic diagram of the mouse skin full-thickness injury model and experiments *in vivo*. A circular wound of 1-cm diameter was made by using ophthalmic scissors on the back of the mouse. The wounds were observed after application of the hydrogel. On days 7 and 14, the skin tissues were taken for follow-up experiments. **(B)** Representative images and schematic diagrams of wounds with different treatments: PBS, PEG hydrogel, and icariin+PEG hydrogel dressings. **(C)** Schematic diagram of the healing process on day 0, 3, 7, 10, 14 after wounding was measured by ImageJ. **(D)** Wound-recovery rates of different treatment groups (control, PEG, and icariin+PEG) over 14 days. *n* ≥ 4. **p < 0.005, ***p < 0.0005, ****p < 0.0001 *vs*. PBS; ^##^ *p* < 0.005, ^####^ *p* < 0.0001 *vs*. PEG. **(E)** HE staining showing the morphological appearance of skin treated with hydrogels on days 7 and 14. The black arrows and broken lines indicate the edge of the wound visible under a microscope. Scale bar: 200 μm.

We used a mouse skin-wound-healing model to evaluate the therapeutic effect of the icariin+PEG hydrogel *in vivo*. The icariin+PEG hydrogel, PEG hydrogel, and PBS were used to treat the injured area, and the wounds at different time points were photographed to investigate the effects of the icariin+PEG hydrogel on the healing rate (Figures 2A,B). A comparison of these images showed that the icariin+PEG group speeded up the wound-healing process compared to that achieved with the other groups (PEG and control groups) (Figure 2B). The wound areas in all groups were clearly reduced after 7 days of treatment; the icariin+PEG group showed the fastest healing compared to others. The changes in the healing process are shown in a composite figure for clarity (Figure 2C). The percentage of the wound area also confirmed the gross observation results (Figure 2D). Subsequently, HE staining was performed to observe wound regeneration (Figure 2E). The control group and the PEG group had a slower closing rate than that achieved with the icariin+PEG group, which is consistent with the results of visual healing. Thick abundant granulation tissue can be clearly seen in icariin+PEG-treated wounds. In contrast, wounds in the control and PEG groups showed a very small amount of newly formed tissue at day 7. Especially on the last day of observation, the regeneration of the wound in the Icariin+PEG group was almost complete, the edges of the wound were not obvious, the thickness of the epidermis was moderate, and the hair follicles in the center of the wound grew well. Hence, it is clear that the icariin+PEG hydrogel enhanced the healing efficacy of wounds and showed a fairly high level of wound recovery.

### Icariin+PEG Hydrogel Suppresses Skin Fibrosis After Wound

**FIGURE 3:**
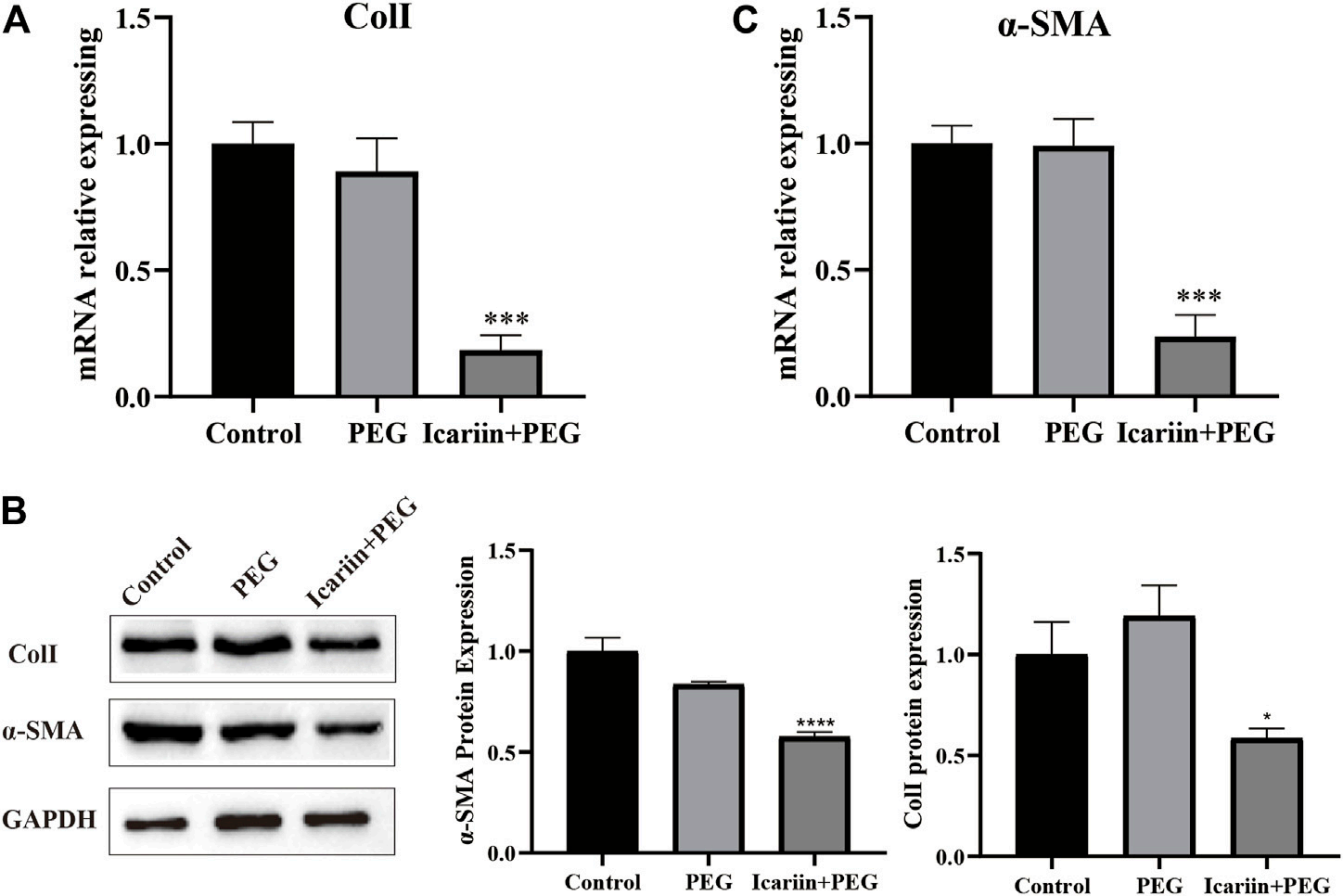
Icariin+PEG hydrogel alleviated skin fibrosis. **(A)** RT-PCR analysis of ColⅠ expression in three groups. **(B)** Protein expression level of Col Ⅰ and α-SMA as detected by western blotting on day 14 at the injured sites. Quantification of the western blotting bands compared to that of the control group. **(C)** RT-PCR analysis of α-SMA expression in three groups.

Wound healing can lead to skin fibrosis, resulting in scar formation and ultimately in the loss of skin functions. Col Ⅰ is the primary material involved in the formation of scar tissue. Thick Col Ⅰ forms large-diameter collagen fibers with horizontal stripes at the wound site, making the scar structure hard and inelastic, which is completely different from the normal skin tissue (Feng et al., 2020; Zhao et al., 2021). On day 14 after treatment, RT-PCR analysis showed that the *Col I* gene expression of the icariin+PEG group was markedly lower than that of the other two groups (Figure 3A). In addition, western blot analysis showed that the icariin+PEG dressing dramatically decreased the protein level of Col I (Figure 3B).

Myofibroblasts proliferate and differentiate into myofibroblasts expressing α-SMA. Subsequently, myofibroblasts continuously migrate and synthesize a large amount of collagen, resulting in excessive fibrosis. Therefore, we used the α-SMA to calculate the number of myofibroblasts in the injured tissue (Limandjaja et al., 2021). Clearly, RT-PCR analysis showed that the α-SMA gene expression of the icariin+PEG group was markedly lower than that of the other two groups (Figure 3C). The western blotting results were consistent with the gene levels measured by PCR (Figure 3B). Taken together, the icariin released from the icariin+PEG hydrogel exerted additional effects by reducing excessive collagen deposition and excess myofibroblast differentiation during skin wound healing, which together promote wound healing.

### Icariin+PEG Hydrogel Promotes M2 Polarization of Macrophages and Enhances Anti-Inflammation at Injured Sites

**FIGURE 4:**
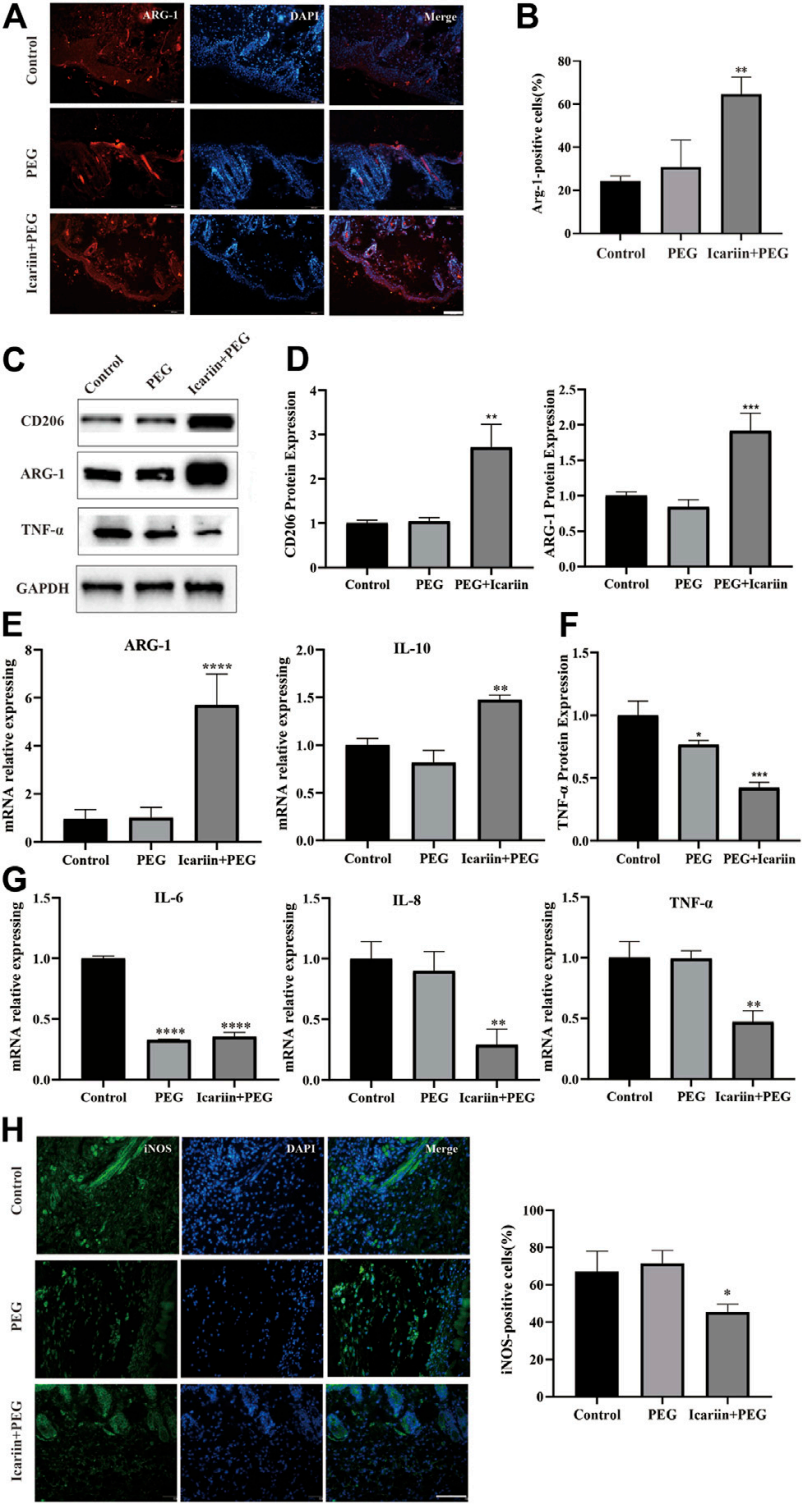
Icariin+PEG hydrogel promoted macrophage M2 polarization and reduced inflammation in the early stage of wound healing *in vivo*. **(A)** Representative immunofluorescence pictures of ARG1 expression on day 7 at the injured sites. Scale bar: 100 μm. **(B)** Quantification of ARG1-positive cells in different groups. **(C,D)** Protein expression level of CD206 and ARG1 as detected by western blotting on day 7 at injured sites. Quantification of the western blotting bands compared to that of the control group. **(E)** Expression levels of M2-related genes *ARG1* and *IL-10* of the three groups by RT-PCR. **(F)** Protein expression level of TNF-α as detected by western blotting on day 7 at the injured sites. Quantification of the western blotting bands compared to that of the control group. **(G)** Expression level of inflammation-related genes IL-6, IL-8, and TNF-α in different groups by RT-PCR. **(H)** Representative immunofluorescence pictures of iNOS expression on day 7 at injured sites. Scale bar: 100 μm. Quantification of iNOS-positive cells in different groups.

As macrophages are the main inflammatory cell type during the early stage of the healing process, their role in both M1 and M2 phenotypic polarization has been extensively studied. M2 phenotype of macrophages has been proved to promote cutaneous wound healing (Zhang et al., 2010). Therefore, we next analyzed the M2 macrophage markers ARG1 and CD206 to explore the distribution of macrophages at the site of the icariin+PEG hydrogel treatment (Kim et al., 2019). As shown in Figures 4A,B, the PEG hydrogel group had fewer ARG1-positive cells; in contrast, their distribution in the icariin+PEG hydrogel group was more widespread under the skin of the wound. This observation indicates that the icariin released by the icariin+PEG hydrogel produced higher levels of M2 macrophages at the wound site (Figures 4C,D). Then, CD206 was chosen as another surface marker of M2 macrophages, and CD68 for macrophages of all the subsets. As shown in the representative fluorescent pictures in Figure S, in the subcutaneous layer on the wounds, the icariin+PEG group exhibited a more widely distribution of CD206 positive cells. ARG1-and CD206-specific antibodies were used to further identify the presence of M2 macrophages during the initial state of the injury. RT-PCR analysis showed that the icariin+PEG hydrogel greatly improved the expression of *IL-10* and *ARG1* genes, which are related to M2 macrophages (Figures 4E,F). Hence, the icariin+PEG hydrogel promoted the polarization of M2 macrophages at the injury site.

Inflammatory cell infiltration initiates the wound-healing process. It removes necrotic material to prepare the wound bed for the subsequent tissue regeneration (Pratsinis et al., 2019). Thus, we explored various anti-inflammatory effects of the different treatment methods using TNF-α as an indicator of the inflammatory response (Arabpour et al., 2021). The early stage of the inflammatory response is characterized by numerous M1 macrophages, which produce large amounts of proinflammatory cytokines. The western blot and PCR results of TNF-α expression showed that the concentration of proinflammatory cytokines in the wound bed was significantly reduced after treatment with the icariin+PEG hydrogel (Figures 4G,H). In addition, the icariin+PEG hydrogel greatly reduced the levels of the local proinflammatory cytokines IL-6 and IL-8 on day 7 after injury (Figure 4G). Furthermore, the inflammatory cell infiltration of the mouse skin injury model was monitored by immunofluorescence to evaluate the role of the icariin+PEG hydrogel on inflammation. The icariin+PEG hydrogel treatment slightly reduced the aggregation of inducible nitric oxide synthase (iNOS)-positive cells (Figure 4I). Hence, the icariin+PEG hydrogel could prevent inflammation at the center of the injury by managing M1 macrophages, thereby reducing the infiltration of inflammatory cells. Therefore, the icariin+PEG hydrogel greatly increased the number of repairing M2 macrophages and improved the quality of wound healing, thus showing important anti-inflammatory effects and repair during the healing process.

### Icariin+PEG Hydrogel Promotes the Regeneration of Hair Follicles at Injured Sites

**FIGURE 5:**
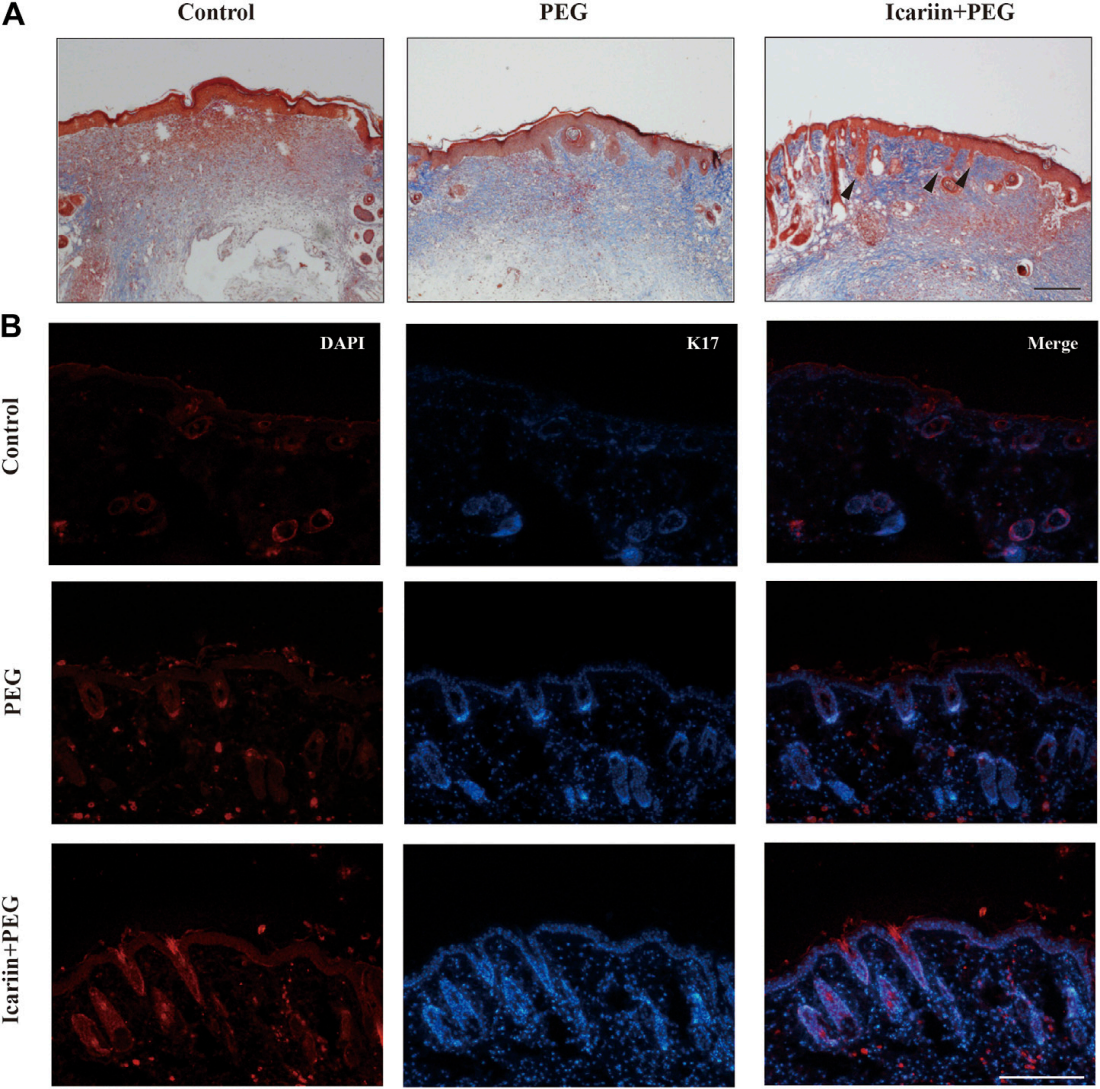
Hair follicles are regenerated with icariin+PEG hydrogel treatment. **(A)** Masson staining of different groups on day 14. Red arrows indicate new hair follicles. Scale bar: 200 μm. **(B)** Representative immunofluorescence images of K17 expression on day 14 at the injured sites. Scale bar: 100 μm.

In addition to the excessive deposition of collagen fibers, another major feature of scarring is the loss of skin accessory organs, especially the aplasia of hair follicles at the late stage of wound healing (Zhang et al., 2016). To further investigate this phenomenon, we investigated hair-follicle regeneration in the skin wound of mice. Apart from severe fibrosis and hyperplasia observed at the wound center, the number of hair follicles was also reduced in the groups treated with PBS or the drug-free hydrogel (Figure 5A). Next, we investigated whether any epidermal cell type promotes epidermal homeostasis and repair in icariin-treated wounds (Ito et al., 2007; Blanpain and Fuchs 2009). Keratin 17 (K17) marks the outer root sheath of specific hair follicles in epidermal cells (Figure 5B). By day 14 after treatment, the tissues treated with the icariin+PEG hydrogel showed an extensive presence of K17 around the epidermis and hair follicles. Although wound contraction may make it difficult to image the complete structure of hair follicles, the presence of epidermal cells in drug-treated tissues is attractive and may increase the possibility of repairing functional hair follicles.

### Icariin+PEG Hydrogel Promotes the Polarization of Macrophages to the M2 Phenotype *in vitro*

Insufficient M2 polarization is related to many pathological processes such as the hard healing surface of chronic wounds, as can be seen in diabetic ulcers, which can inhibit the inflammatory response and hinder tissue regeneration (Ferrante and Leibovich 2012). Therefore, we first tested the biological effects of icariin on the polarization of THP-1 macrophages *in vitro* on the M2 phenotype. After 24 h of treatment with the polymethacrylate (PMA) copolymer, the THP-1 macrophages that adhered to the wall from a suspended state successfully differentiated into M0 macrophages (Supplementary Figure S3A). Flow cytometry experiments revealed an increase in the expression of CD68 induced by PMA, further proving the formation of M0 macrophages (Supplementary Figure S3B).

**FIGURE 6:**
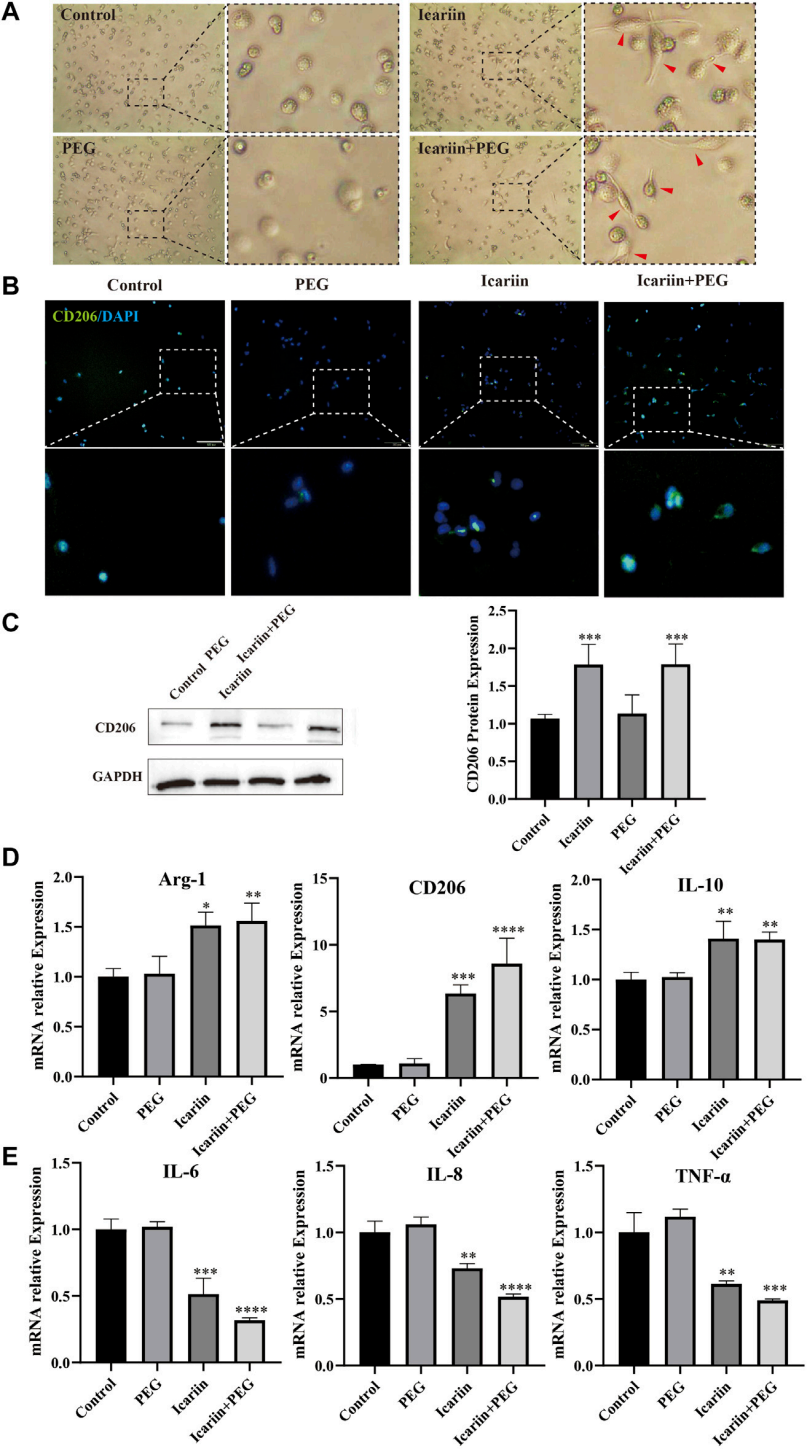
Icariin+PEG hydrogel promoted M2 polarization of macrophages and enhanced anti-inflammatory effects *in vitro*. **(A)** Morphological changes of THP-1 macrophages after icariin treatment for 48 h were observed under an optical microscope. Red arrows indicate elongated macrophages. **(B)** Representative pictures of THP-1 immunofluorescence staining with CD206 antibody after being co-cultured with the hydrogel for 48 h. Scale bar: 100 μm; scale bar of magnified pictures: 25 μm. **(C)** Western blot analysis of the CD206 protein expression level in macrophages treated with different dressings for 48 h. Quantification of the western blotting bands compared to that of the control group. **(D)** RT-PCR analysis of M2-related gene (*ARG1*, *CD206*, and *IL-10*) expression in macrophages. **(E)** RT-PCR analysis of M1-related gene (*IL-6*, *IL-8*, and *TNF-α*) expression in macrophages.

Next, we cultured the newly formed M0 macrophages with PBS, icariin, PEG hydrogel, icariin+PEG hydrogel in the upper chamber of the transwell insert. The macrophages were treated with different dressings for 48 h. Figure 6A shows that the macrophages in an environment without icariin are round and aggregated. In contrast, longer spindle-shaped macrophages formed in the presence of icariin. Likewise, the icariin+PEG hydrogel-treated macrophages showed a significantly higher average fluorescence intensity of *CD206* staining, indicating that the M2 polarization state of macrophages had increased considerably (Figure 6B). In addition, western blotting showed a higher level of CD206 protein, further confirming the effect of icariin+PEG hydrogel on the polarization of macrophages (Figure 6C). These results are consistent with the expression of M2 macrophage-related genes (*IL-10*, *CD206*, and *ARG1*) after treatment with the icariin+PEG hydrogel for 48 h (Figure 6D). We further verified the polarization of M2 macrophages after icariin treatment by flow cytometry (Supplementary Figure S4). Specifically, compared with the mean fluorescence intensity (MFI) for CD206 staining of the control (0.27), the MFI of the icariin group was significantly increased to 32.4, while that of the positive control group was 41.8. Hence, icariin induced the differentiation of macrophages to the M2 anti-inflammatory type. Additionally, the icariin+PEG hydrogel inhibited the expression of M1 macrophage-related genes (*IL-6*, *TNF-α*, and *IL-8*) after 48 h of treatment (Figure 6E). Therefore, the icariin+PEG hydrogel increased the number of M2 anti-inflammatory macrophages even in the inflammatory microenvironment *in vitro*. Furthermore, icariin regulated the polarization of M2 macrophages involved in wound healing.

### Icariin+PEG Hydrogel Facilitates the Migration, Viability, and Gene Expression of Hair Follicle Dermal Papilla Cells

**FIGURE 7:**
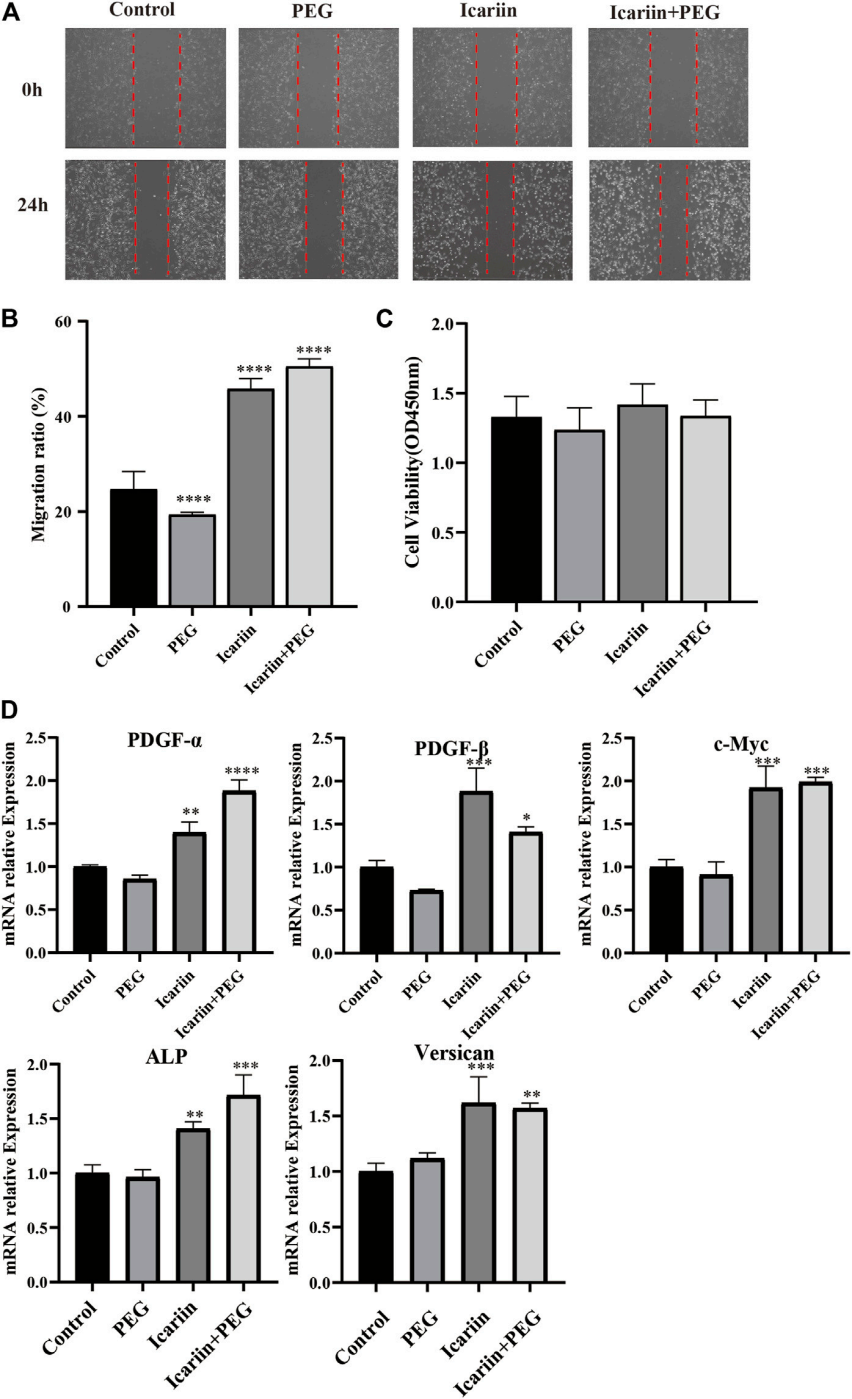
Effect of icariin+PEG hydrogel on the migration, viability, and gene expression of hair follicle dermal papilla cells (HHDPCs). **(A)** Effect of icariin+PEG hydrogel on the migration of HHDPC cells. **(B)** Migration rate of HHDPCs. **(C)** Biocompatibility of different groups. **(D)** RT-PCR analysis of genes that can promote hair-follicle regeneration (*PDGF-α*, *PDGF-β*, c-*Myc, ALP, and Versican*).

Hair dermal papilla is located at the base of the hair follicle and produces hair fibers by inducing epidermal hair-follicle development (Kwack et al., 2018). It plays a crucial role in the hair growth cycle, which is regulated by various molecular pathways, including BMP4 signaling (Liu et al., 2021). To further investigate the effect of icariin+PEG hydrogel, HHDPCs were used to co-culture with separate PEG, separate icariin, and icariin+PEG hydrogel. Icariin alone as well as the icariin+PEG hydrogel effectively promoted HHDPC migration (Figures 7A,B). Furthermore, we compared the cell numbers with the results of CCK8 analysis after 48 h (Figure 7C). No significant difference was observed between different groups, which proved that our novel material did not harm the vitality and proliferation of HHDPCs. In addition, an obvious effect of the icariin+PEG hydrogel combination in stimulating the expression of some cytokines that can promote hair-follicle regeneration was observed (Figure 7D). Significantly, the icariin+PEG hydrogel could stimulate the expression of *PDGF-α*, *PDGF-β*, and *c-Myc* (genes of hair-follicle anagen markers), indicating the stimulation of hair-follicle growth (Kamp et al., 2003; Dong et al., 2017).

### BMP4 Signaling Is Required for the Effects of icariin+PEG Hydrogel on Macrophages and HHDPCs

**FIGURE 8:**
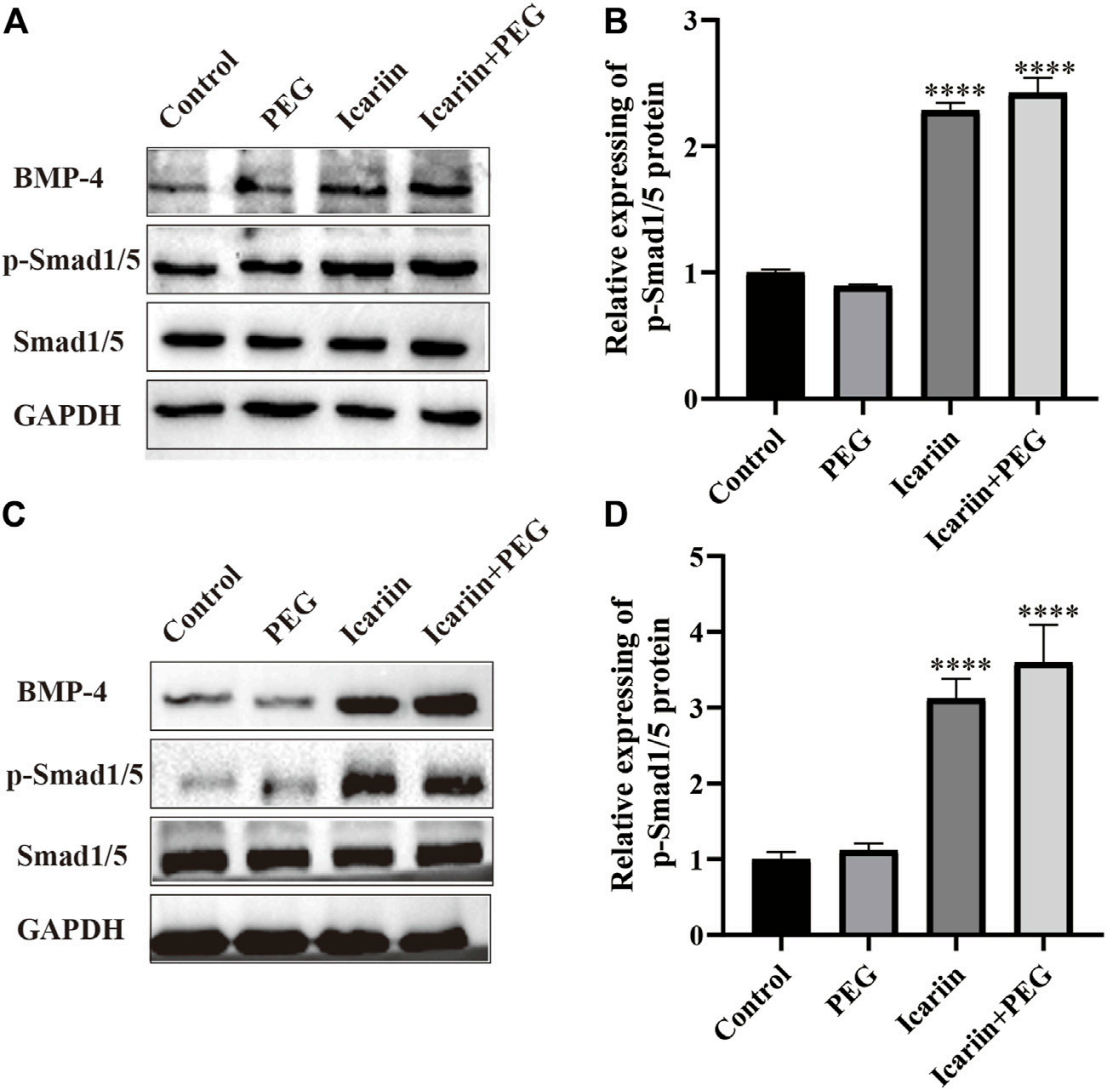
Participation of BMP4 in M2 macrophage polarization and hair-follicle regeneration. **(A)** Protein expression levels of BMP4, p-Smad1/5, and Smad1/5 as detected by western blotting in the macrophages treated with the different dressings for 48 h. **(B)** Quantification of western blotting bands. **(C)** Protein expression levels of BMP4, p-Smad1/5, and Smad1/5 as detected by western blotting in the HHDPCs treated with the different dressings for 48 h. **(D)** Quantification of western blotting bands.

BMP4 plays an important role in embryonic development and tissue homeostasis (Botchkarev and Sharov 2004; Tong et al., 2015; Zylbersztejn et al., 2018). Previous work has shown that icariin is associated with the activation of BMP signaling (Sen et al., 2009; Singh et al., 2019). To determine whether icariin+PEG hydrogel-induced BMP signaling was required for macrophage M2 polarization during the wound healing process, we analyzed western blotting results and noted that BMP4 and the downstream phosphorylated Smad1/5 expression were observably increased after the addition of the icariin+PEG hydrogel (Figures 8A,B). Meanwhile, the BMP4 and p-Smad1/5 protein expression of HHDPCs treated with icariin+PEG hydrogel was significantly increased compared to that achieved with the control group (Figures 8C,D). Moreover, our immunofluorescence staining of the skin wound revealed that most hair follicles exhibited the activation of Smad1/5 phosphorylation at the dermal papilla, in contrast to the controls (Supplementary Figure S5). These results suggest that the icariin+PEG hydrogel can activate BMP4 signaling, which promotes macrophage M2 polarization and hair-follicle regeneration, thus increasing the possibility of wond repair.

## Discussion

**SCHEME 1:**
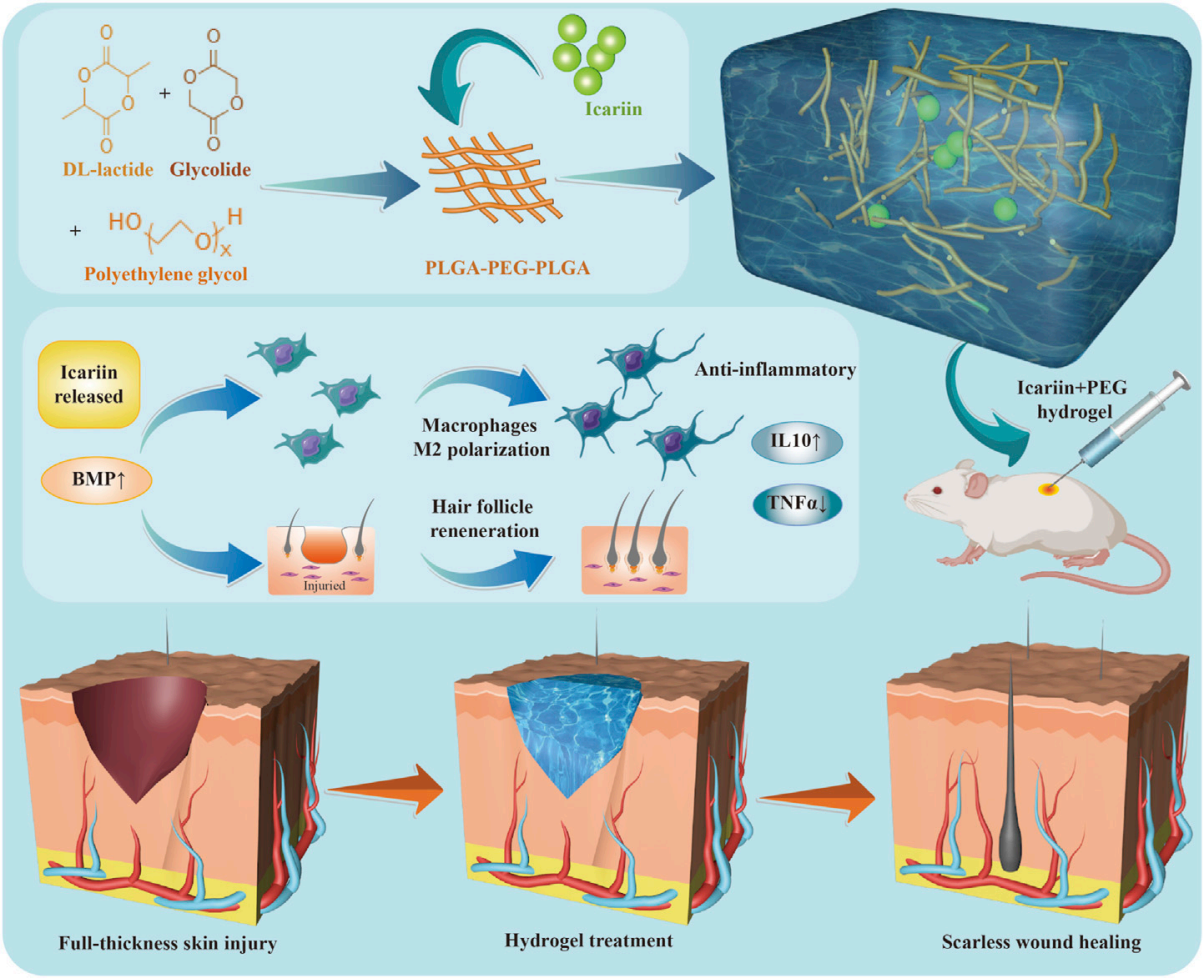
Formation of icariin+PEG hydrogels and mechanism of wound healing.

This study demonstrates for the first time that a thermosensitive hydrogel can adapt to wounds of different shapes and gradually release icariin to further promote wound healing. The study also shows that icariin+PEG hydrogels can polarize macrophages towards an M2 anti-inflammatory phenotype during the wound healing process. In addition, this study shows that the icariin+PEG hydrogel was involved in regulating the formation of new hair follicles and speeding up the process of wound healing. At the molecular level, the icariin+PEG hydrogel promoted wound healing by inducing BMP4 and downstream phosphorylated Smad1/5 activation. A schematic representation of this study is depicted in Scheme 1.

The typical treatment for full-thickness skin trauma involves skin transplantation, including autologous transplantation, allogeneic transplantation, xenotransplantation, and skin substitutes (Tavis et al., 1978). Skin substitutes include synthetic scaffolds that can be made with three-dimensional bioprinting using cells (Hosseini and Shafiee 2021). Many bioactive dressings have been developed. In many of these dressings, substances conducive to wound healing can be added, such as drugs, cytokines, and growth factors (Kaushik et al., 2015). A hydrogel is a three-dimensional hydrophilic polymer network, whose structure and function are similar to those of natural extracellular matrix (Sun et al., 2018). In addition, the hydrogel can protect these substances without changing their properties, which can then be smoothly delivered to related cells (Xu et al., 2018). PEG-based polymers have a superior biocompatibility; hence, they have been studied as wound-healing scaffolds (Shi et al., 2021). Our team developed a PEG-based temperature-sensitive hydrogel that can be formed *in situ*. The hydrogel had good injection ability and could be injected with a needle to adapt to any irregular shape (Supplementary Figure S1A). The time required for turning the liquid hydrogel into a gel could be managed by temperature, which makes the treatment and care of irregular wound more convenient. Rheological tests showed that the hydrogels could fill any irregular wounds before the sol–gel transition process and had excellent self-healing properties. Notably, realization of repair of the complete skin structure mainly depends on the optimum combination of cells, signaling molecules, and scaffolds.

During wound care, any imbalance or defect in the coordinated interaction between multiple cell types, cytokines, and the extracellular matrix can disrupt the balance of related cells and make it difficult to achieve optimum tissue repair, leading to hypertrophic scars that further impair normal tissue function and eventually lead to organ failure and death (Wu et al., 2019). Interestingly, scarless healing can be seen in human fetuses; those with a cleft lip and palate have no obvious scars after undergoing laparoscopic repairs (Mast et al., 1992; Longaker et al., 1994; Bartkowska and Komisarek 2020). Comparison of the wound-healing process in adults and fetuses shows that fetal wounds are characterized by milder inflammation (Lin et al., 1994; Liechty et al., 2000; Walmsley et al., 2015). While the inflammatory response is essential to protect the body from infection and necrosis, preparing the body for the subsequent regeneration is also a major pathogenic factor in scar formation (Childs and Murthy 2017). Fibroblasts, the most important cells involved in repairing damaged tissues, are closely related to inflammation (Landén et al., 2016; McGinty and Siddiqui 2021). When there are excessive inflammatory cells in the early stage of wound healing, fibroblasts are more likely to differentiate into myofibroblasts under the influence of proinflammatory factors like TNF-α and IL-6 (Thulabandu et al., 2018).

Macrophages are widely distributed and one of the most important immune cells in wound healing (Arabpour et al., 2021; Wolf et al., 2021). They are the main phagocytes that perform clearance and secretion functions during the inflammatory phase of wound healing. The phenotype of macrophages and the types of cytokines released can change at different times after trauma and are closely related to the inflammation and remodeling stage of the wound (Zhang and Mosser 2008; Martinez et al., 2009). They can be stimulated into different types with contrasting functions. Several studies have shown that macrophages undergo morphological changes under the stimulation of different molecular signals (Luu et al., 2015; Jia et al., 2019). In contrast to the M1 type, anti-inflammatory macrophages tend to be elongated (Porcheray et al., 2005; Gao et al., 2019). The morphological changes of macrophages are related to their functional polarization (McWhorter et al., 2015). Elongated macrophages tend to polarize into M2 macrophages and inhibit M1 polarization. We verified that macrophages were induced to polarize to the elongated anti-inflammatory M2 phenotype by icariin released from the hydrogel both *in vivo* and *in vitro*. Furthermore, we showed that the icariin+PEG hydrogel accelerated wound healing and reduced tissue fibrosis. However, it should also be investigated whether persistently inhibiting M1 macrophages will make wounds susceptible to infection and thus difficult to heal, forming chronic wounds. Considering the dual role of macrophages in the wound-healing process, it is necessary to carefully explore the balance between proinflammatory and anti-inflammatory cells.

If the hair follicles are completely destroyed, they cannot be fully regenerated and scar repair occurs (Boyce and Lalley 2018). Several different progenitor cell populations in hair follicles, including bulge, upper bulge, tight junction barrier in hair follicles, and the infundibulum next to sebaceous glands, play an important role in skin regeneration (Hu et al., 2014; Mokos and Mosler 2014; Garcin and Ansell 2017). Genetic tracing of epithelial stem cells expressing K17 in the germ area of the secondary hair of the hair follicle showed that these cells migrated to the epidermis from the full-thickness outer wound surface. This observation showed that after trauma disrupts the epidermal homeostasis, the epithelial stem cells in hair follicles proliferate, supplement epidermal cells, and promote re-epithelialization. Fortunately, our research showed that additional new hair follicles would appear on the skin with this new medicated hydrogel. Hence, it can be speculated that the use of the icariin+PEG hydrogel could exert the expected therapeutic influence. Furthermore, immunofluorescence experiments indicated that most of the hair follicles showed Smad1/5 phosphorylation activation at the dermal papilla, strongly suggesting that the icariin+PEG hydrogel stimulated the formation of hair follicles by activating the BMP4 pathway.

Although BMP signaling has not been directly linked to any stage of wound healing, BMPs are a promising option for skin regeneration because they are related to hair-follicle regeneration and might promote wound healing (Plikus et al., 2017). Interestingly, BMP4 resulting from acute lymphoblastic leukemia can upregulate the expression of interleukin (IL)-10 and promote the polarization of M1-like macrophages to the M2 phenotype (Zylbersztejn et al., 2018). Our results also confirmed that BMP4 signaling is required for the effects of icariin+PEG hydrogel on macrophages and HHDPCs. However, BMP signaling of other cell types in response to injury has not been studied. Previous studies have proved a BMP5 gene regulates the injury element in zebrafish wound models; however, the mechanisms that lead to the reactivation of BMPs following injury are still unknown (Heller et al., 2022). Therefore, further studies on the therapeutic effects of BMP signaling should be performed to investigate whether it can be targeted therapeutically in skin fibrotic diseases.

## Conclusion

Our group developed a new thermosensitive *in situ* injectable hydrogel. The precursor hydrogel could be injected into wounds of any shape. It could quickly become a colloid when heated to the temperature of the skin surface, with good mechanical properties and biocompatibility. *In vitro*, the icariin in the hydrogel enhanced the BMP4 signaling pathway and promoted the conversion of macrophages from the M1 phenotype to the M2 phenotype. *In vivo*, the hydrogel reduced inflammation in the early stage of wound healing and significantly increased the number of new hair follicles in the late stage. Overall, the icariin released from the icariin+PEG hydrogel reduced excessive collagen deposition and excess myofibroblast differentiation during skin-wound healing, thereby promoting healing and hair-follicle regeneration. Therefore, it has great potential for practical applications. However, any future successful clinical application of icariin+PEG hydrogels depend on optimizing the release of icariin and precisely regulating the spatiotemporal molecular signals at the injured site during wound healing.

## Data Availability

The datasets presented in this study can be found at https://figshare.com/articles/dataset/Date_zip/20138330, DOI:10.6084/m9.figshare.20138330.
