# Supplementary material for: Dual-Action Icariin-Containing Thermosensitive Hydrogel for Wound Macrophage Polarization and Hair-Follicle Neogenesis
Source: Front Bioeng Biotechnol. 2022 Jun 27;10:902894. doi: 10.3389/fbioe.2022.902894 (PMC9272914; doi:10.3389/fbioe.2022.902894)
Supplement: Supplementary file 1 [file DataSheet1.docx]

**Supplementary Materials**

**Table S1:** Primers used in RT-PCR.

| Transcription | Prime sequences |
| --- | --- |
| CD206 | Forward: TTCAGTGGACCATCGAGGAAGAGG  Reverse: ATGGCAACACACCCTGGCTTTC |
| IL-10 | Forward: GCCAAGCCTTGTCTGAGATGATCC  Reverse: GCCTTGATGTCTGGGTCTTGGTTC |
| Arg-1 | Forward: TCAAAGGGACAGCCACGAGGAG  Reverse: GGATGTCAGCAAAGGGCAGGTC |
| Col1 | Forward: TGGCAAAGAAGGCGGCAAAGG  Reverse: AGGAGCACCAGCAGGACCATC |
| TNF-α | Forward: TGGAGAAGGGTGACCGACTCAG  Reverse: TCCCAAAGTAGACCTGCCCAGAC |
| IL-6 | Forward: GGTGTTGCCTGCTGCCTTCC  Reverse: GTTCTGAAGAGGTGAGTGGCTGTC |
| IL-8 | Forward: GGACCACACTGCGCCAACAC  Reverse: CCCTCTGCACCCAGTTTTCCTTG |
| α-SMA | Forward: TCGTGCTGGACTCTGGAGATGG  Reverse: GAAGGAATAGCCACGCTCAGTCAG |
| c-Myc | Forward: AGCAGCGACTCTGAGGAGGAAC  Reverse: TCCAGCAGAAGGTGATCCAGACTC |
| PDGFα | Forward: GGATTCTTTGGACACCAGCCTGAG  Reverse: CGATGCTTCTCTTCCTCCGAATGG |
| PDGFβ | Forward: TCTCTGCTGCTACCTGCGTCTG  Reverse: AAGGAGCGGATCGAGTGGTCAC |
| ALP | Forward: GCCTACACGGTCCTCCTATACGG  Reverse: CACTGCTGACTGCTGCCGATAC |
| Versican | Forward: CCATCTCACAAGCATCCTGTCTCAC  Reverse: CTGCCATCAGTCCAACGGAAGTC |
| GAPDH | Forward: TCCGTGTCCCCACTGCCAAC  Reverse: CGCCTGCTTCACCACCTTCTTG |

**Supplementary Figures**


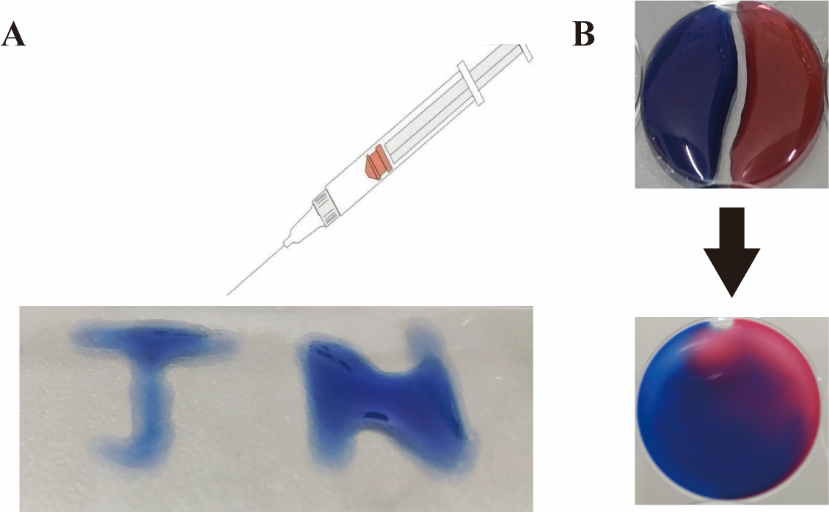


**Fig. S1. Injectability and self-healing properties of the PEG hydrogel. (A)** Morphological changes of THP-1 macrophages at 24 h after PMA treatment as observed under an optical microscope. **(B)** Representative flow cytometry dot plots. CD68-positive macrophages were subjected to flow cytometry.


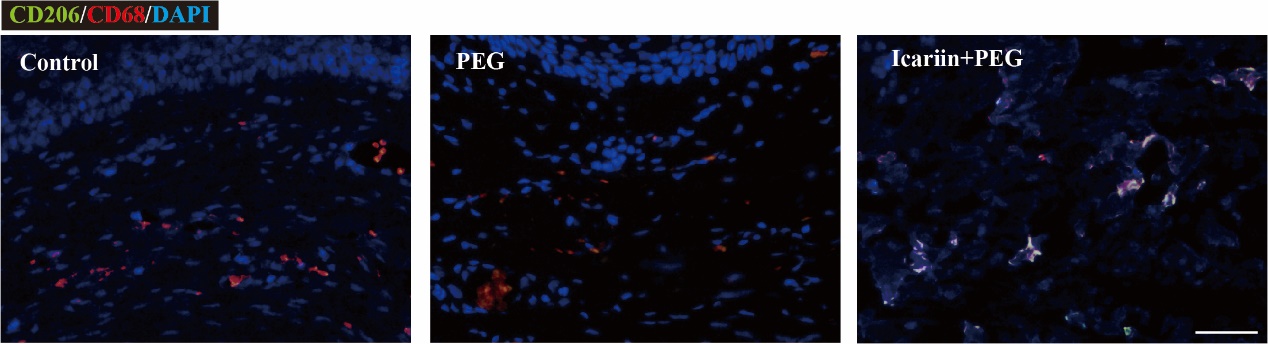


**Fig. S2.** **Representative immunofluorescence pictures of CD206 expression on day 7 at the injured sites. Scale bar: 50 μm.**


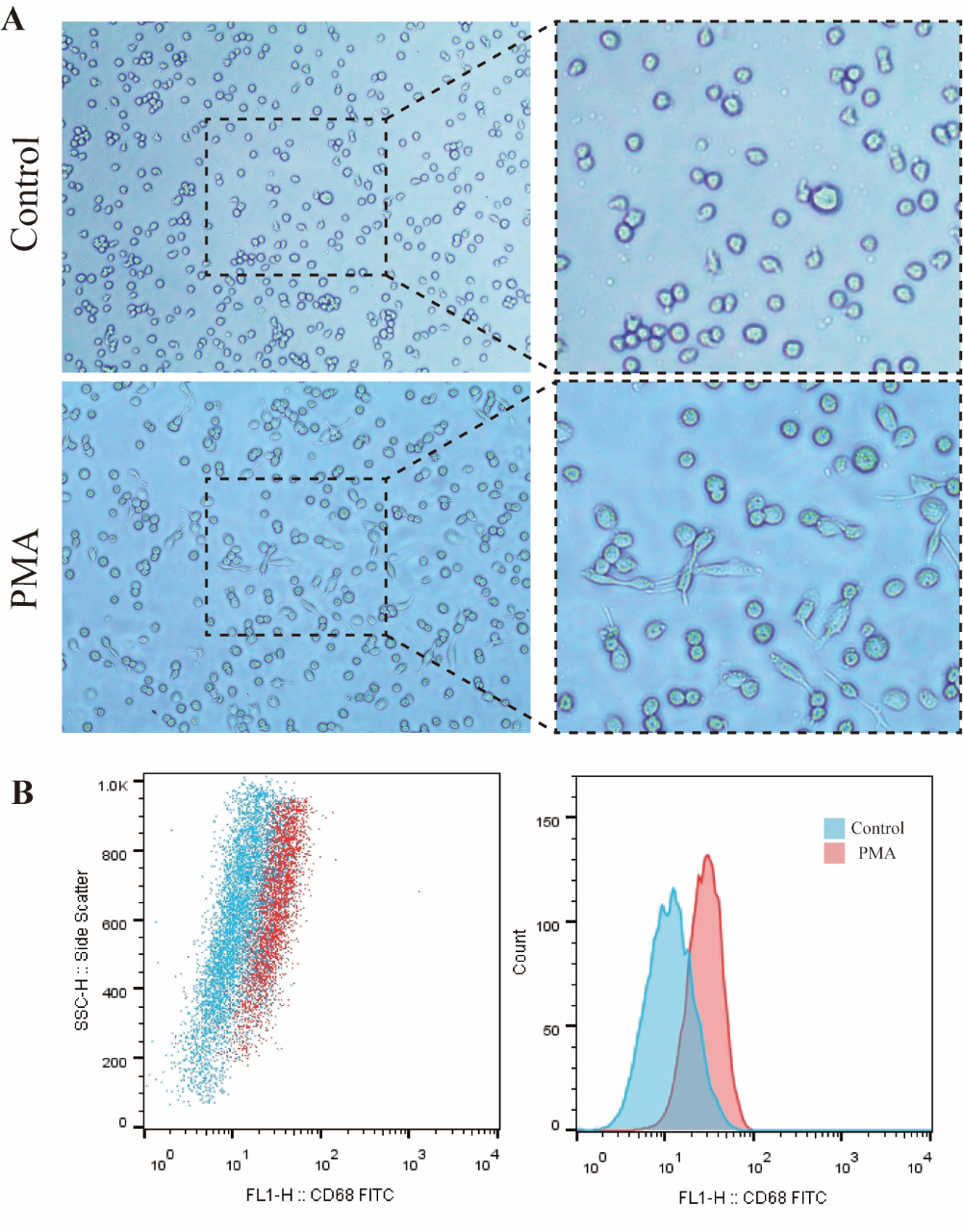


**Fig. S3. (A)** Morphological changes of THP-1 macrophages at 24 h after PMA treatment as observed under an optical microscope. **(B)** Representative flow cytometry dot plots. CD68-positive macrophages were subjected to flow cytometry.


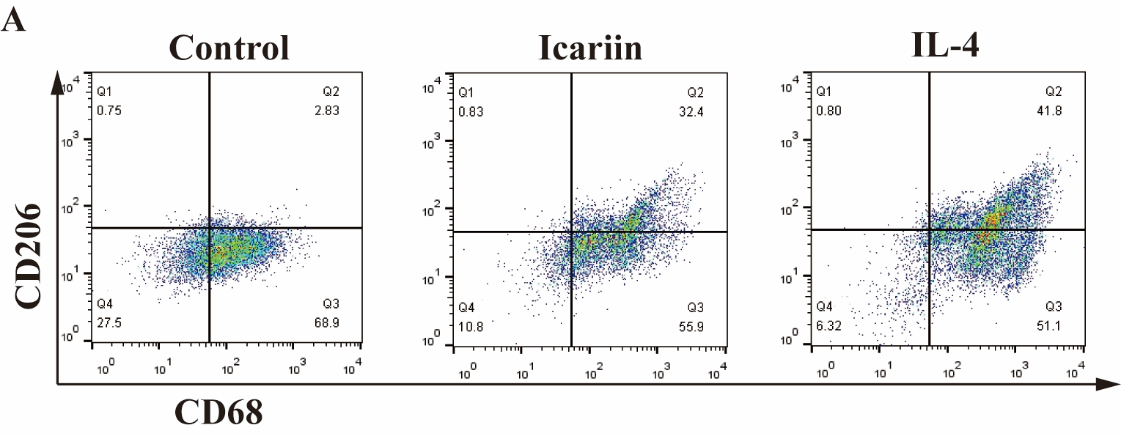


**Fig. S4. Treatment with the icariin+PEG hydrogel reduced inflammation *in vitro*. (A)** Representative flow cytometry dot plots. Macrophages double stained with CD68 and CD206 were subjected to flow cytometry after treatment for 48 h. The IL-4 group served as a positive control.


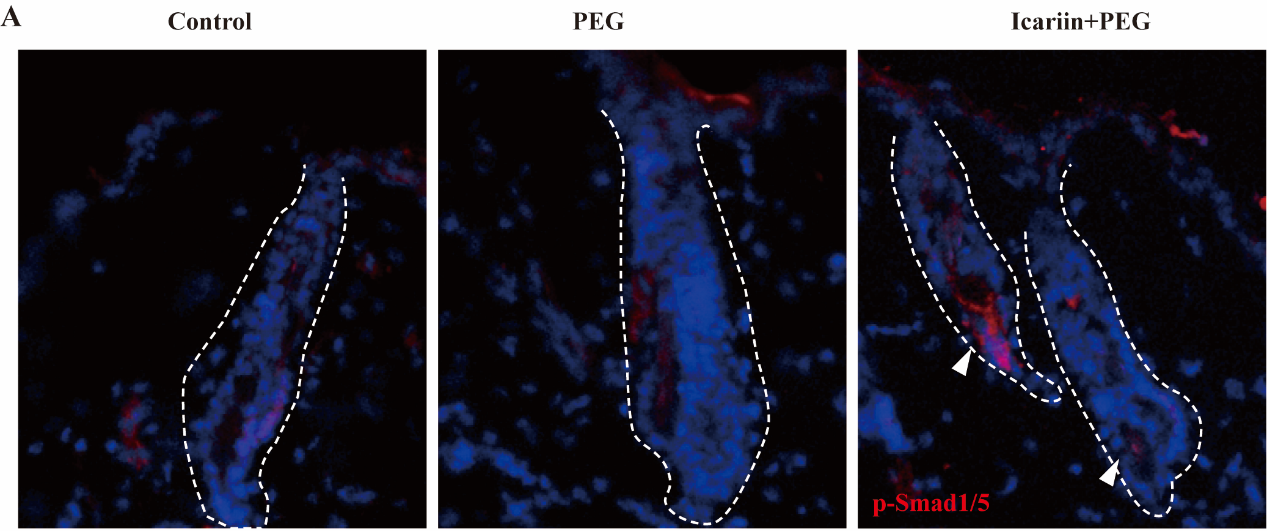


**Fig. S5. Icariin+PEG hydrogel activated BMP4 signaling to promote hair-follicle regeneration. (A)** Representative immunofluorescence pictures of p-Smad1/5 expression on day 14 at the injured sites. The white arrows indicate the expression of p-Smad1/5, while the white dotted lines indicate hair follicles of the skin.
